# Face perception in the Japanese population using EEG

**DOI:** 10.3389/fpsyg.2025.1555645

**Published:** 2025-10-22

**Authors:** Kensaku Miki, Yasuyuki Takeshima, Shoko Watanabe, Ryusuke Kakigi

**Affiliations:** ^1^School of Nursing, University of Human Environments, Obu, Aichi, Japan; ^2^Department of Integrative Physiology, National Institute for Physiological Sciences, National Institutes of Natural Sciences, Okazaki, Aichi, Japan; ^3^Higashi Owari National Hospital, National Hospital Organization, Nagoya, Aichi, Japan

**Keywords:** electroencephalography (EEG), N170, face, Japanese population, Western populations

## Abstract

The face contains abundant information and plays an important role in our interactive communication. Electroencephalography (EEG) has excellent temporal resolution, making it a useful tool for investigating the time-sequence of face perception processes. Studies on development, conducted in Western populations, found that event-related potential (ERP) changes in children begin in mid-childhood. However, there are few studies on developmental changes in Japanese children. In addition, few studies have investigated whether hospitality expertise affects these processes in Western and Japanese populations. In this review, we summarize evidence from EEG studies that investigated face perception processes, with a focus on three developmental and expertise-related studies with Japanese participants. These findings are compared with studies involving Western participants to explore the influence of cultural and experiential factors on face-related brain responses. The face detection pattern in 13-year-old Japanese children is similar to that in adults. This suggests that face detection matures at this age in the Japanese population, differing from findings in Western populations. In addition, few studies have investigated the face emotional change perception process in Western and Japanese populations. However, ERP patterns in response to facial emotional changes in Japanese children aged 7–14 differs from that in adults. This suggests that the process of perceiving facial emotional changes in the Japanese population does not fully mature by the age of 14. Moreover, facial emotion perception in the Japanese population may be influenced by hospitality expertise. We propose the following hypotheses based on this review: (1) the age of maturation for face detection and facial emotional change perception processes are different, (2) expertise may increase attention to emotion and affect the early stage of the face perception process due to training and experience, and (3) the face perception process in the Japanese population differs from that in Western populations.

## Introduction

The human face plays a crucial role in social communication, as it conveys a wide range of information. We perceive information, such as whether a person is familiar to us, when we interact with others. Facial information includes identity, expression, and speech. The classic model of the face perception process (Bruce and Young, 1986) suggests that the processes of facial identity and expression perception are independent.

A distributed human neural system for face perception, aligning with the classic face model, has been suggested (Haxby et al., 2000; Ishai et al., 2005; Ishai, 2008). The classical model contains two visual pathways related to facial information analysis (Haxby et al., 2000). One pathway is related to facial changeable information, such as expression, gaze, and lip movement, and involves the superior temporal sulcus (STS), whereas the other is related to facial changeless information, such as facial identity, and involves the lateral fusiform gyrus (Calder and Young, 2005).

Non-invasive human brain measuring methods, such as functional magnetic resonance image and magnetoencephalography, are useful for investigating human brain function. There have been many studies using these methods to examine face perception processes.

Electroencephalography (EEG), with its excellent temporal resolution, is useful for investigating the time-sequence of face perception processes. In previous EEG studies on normal adults, a negative component was evoked approximately 170 ms after presentation of a face at each electrode in the right and left temporal areas, especially the right, which was termed N170 (George et al., 1996; Bentin et al., 1996). The amplitude of N170 for a face is larger than that for an inanimate object, such as a car or chair (Rossion and Jacques, 2008); therefore, N170 is thought to be related to face perception. In addition, the latency of N170 for eyes is longer than that for a face (Bentin et al., 1996, Taylor et al., 2001a, b, Shibata et al., 2002, Itier et al., 2006, 2007, 2011, Kloth et al., 2013, Nemrodov et al., 2014, Nemrodov and Itier, 2011); therefore, it is related to the process of quickly detecting a face.

A previous psychological study (Farah et al., 1995) suggested that it is more difficult to detect an inverted face than an upright face. This phenomenon is termed the “face inversion effect.” Previous studies reported that N170 is longer for an inverted face than an upright face (Rossion et al., 1999, 2000; Itier and Taylor, 2002; Sadeh and Yovel, 2010). As the face perception process is effective for detecting an upright face in adults, N170 serves as a biomarker of mature face perception.

In EEG studies of visual perception, a positive component begins 90–120 ms at the right and left occipital areas after the presentation of various visual stimuli (Bentin et al., 1996; Allison et al., 1999), which is termed P100. P100 is thought to be related to the early processing of visual perception. It is sensitive to luminance, luminance contrast, and size stimuli but not to the category of the stimulus (Allison et al., 1999). In addition, P100 is affected by visual attention (Mangun, 1995; Hillyard et al., 1998).

P100 is also related to face perception, and is modulated by information of the face, such as gender, ethnicity, and emotion. Dennis et al. (2009) reported that P100 latency is shorter for a fearful face than for a sad face. Moreover, P100 emotion and face effects are strongly influenced by low-level information (Schindler et al., 2021).

A positive component that begins 300–500 ms after a presented stimulus at the parietal areas evoked by selective attention is termed P300 (P3). In the oddball task, participants count how often target stimuli appear among standard stimuli. The target stimuli induce P300 (Jeon and Polich, 2001). In previous studies, attention to angry or fearful faces increases P300 amplitude (Rossignol et al., 2005; Kiss and Eimer, 2008; Lin et al., 2020).

In this review, we summarize evidence from EEG studies that investigated face perception processes, with a focus on three developmental and expertise-related studies with Japanese participants (Miki et al., 2011, 2015, 2022). These findings are compared with studies from Western participants to explore possible cultural and experiential influences on face-related brain responses. In this review, we cited studies retrieved from PubMed using the keywords ‘face,’ ‘EEG,’ ‘N170,’ ‘P100,’ ‘development,’ and ‘expertise.’ Studies were included based on the following criteria: published in English and full-text availability. Herein, we discuss the effect of face perception processes with development and expertise for the Japanese population compared with Western populations.

## Face perception in typically developing Japanese children

Many previous studies investigated developmental changes in face perception processes for infants (Taylor et al., 2004; Melinder et al., 2010; Peykarjou and Hoehl, 2013; Xie et al., 2019; Safar and Moulson, 2020) and children (Meaux et al., 2014; Batty and Taylor, 2006; Vlamings et al., 2010; Kamps et al., 2022). These studies used EEG because it allows for relatively unrestricted body movements during recording.

An EEG study that focused on N170 changes with development in children found that inversion produced latency and amplitude effects on N170 starting in mid-childhood (Taylor et al., 2004). In addition, infant studies of face perception reported that a component termed N290, corresponding to adult N170, was seen in posterior electrodes within 290–350 ms after face stimulus onset in 3–12-month-old children (de Haan et al., 2003; Halit et al., 2003). N290 showed increased sensitivity to faces compared with other kinds of visual stimuli (Halit et al., 2003), and its amplitude was modulated by the face inversion effect in infants (Halit et al., 2003; Peykarjou and Hoehl, 2013).

Few EEG studies have examined the changes in face perception in typically developing Japanese children, compared with many studies in typically developing Western children. We previously investigated N170 changes related to the face detection process in typically developing Japanese children (Miki et al., 2015). Our study focused on the process of detecting an upright face faster than an inverted face and eyes. We investigated 82 typically developing Japanese children, who were divided into six age groups: 8-year-olds (*n* = 11), 9-year-olds (*n* = 17), 10-year-olds (*n* = 15), 11-year-olds (*n* = 12), 12-year-olds (*n* = 10), and 13-year-olds (*n* = 17). Three stimuli were presented to the participants: (1) upright face: upright images of a neutral face, (2) inverted face: inverted images of an upright face, and (3) eyes: eyes alone. Event-related potentials (ERPs) were obtained by averaging EEG signals across trials. EEG electrodes were placed at Fz, Cz, T3, T4, C3, C4, Pz, P3, P4, T5, T6, O1, and O2 as well as T5’ (2 cm below T5) and T6’ (2 cm below T6), according to the international 10–20 system. The reference electrode was placed on the tip of the nose. For the ERP analysis, the time window for the analysis ran from 100 ms before to 400 ms after stimulus onset, and the data obtained during the 100 ms before stimulus onset were used as the baseline. For artifact rejection, epochs in which the variations in the EEG and electrooculogram (EOG) signals were larger than ± 80 μV were automatically excluded from the online averaging.

N170 peak latency significantly decreased with age and was the shortest for the 13-year-old children with significant differences among all three stimulus conditions. N170 peak latency was the shortest for the upright face and the longest for the eyes, which differed from the 8–12-year-old children. This study of typically developing Japanese children demonstrates that the N170 pattern resembles the adult form by age 13. This suggests that the process of face detection matures at 13 years old in the Japanese population.

Emotion is a type of facial information that is important in social interaction. In particular, facial changes reflect changes in emotion and sympathy. Several ERP studies on the facial emotional perception process in face perception used static faces. In adults, N170 is modulated by positive and/or negative emotions (Hinojosa et al., 2015). There have also been several studies on ERP changes related to the facial emotional perception process as the child develops. In a previous study of children, the adult pattern of N170 related to emotions was only observed from the age of 14–15 years old (Batty and Taylor, 2006).

In infants, N290 for fearful faces was significantly smaller (less negative) than that for happy faces in 3-month-old infants (Safar and Moulson, 2020), and N290 was significantly larger for fearful faces than for angry faces in 7-month-old infants (Hoehl and Striano, 2008). On the other hand, a study of 7-month-old infants did not find that emotion had an effect (Leppänen et al., 2007).

These ERP studies on facial emotion perception processes used static faces; however, few ERP studies have examined facial emotional changes. We hypothesized that facial emotional change stimuli may also be important in the distributed human neural system for face perception (Haxby et al., 2000); thus, we investigated the facial emotional perception process instead of static face. We investigated ERP changes related to the facial emotional changes perception process in face perception using facial emotional changes in typically developing Japanese children (Miki et al., 2011). The study included 68 typically developing Japanese children consisting of 39 younger children (23 females, 16 males; 7–10 years old) and 29 older children (10 females, 19 males; 11–14 years old), and 12 typically developed Japanese adults (6 females, 6 males; 23–33 years old). We presented the following four conditions where the first stimulus (S1) was replaced by a second stimulus (S2) with no inter-stimulus interval. Participants perceived each of the following emotional changes: (1) from neutral face to happy face (N-H): appearance of positive emotion, (2) from happy face to neutral face (H-N): disappearance of positive emotion, (3) from neutral face to angry face (N-A): appearance of negative emotion, and (4) from angry face to neutral face (A-N): disappearance of negative emotion. ERPs were recorded by averaging EEG signals across trials. EEG electrodes were placed at FCz, F3, F4, Fz, C3, C4, Cz, T3, T4, T5, T6, P3, P4, Pz, O1, and O2 as well as T5’ (2 cm below T5) and T6’ (2 cm below T6), according to the 10% extension of the international 10–20 system. The reference electrode was placed at the tip of the nose, far from the temporal area. The time frame of averaging was from 100 ms before and 500 ms after S2 onset, with 100 ms before S2 onset used as the baseline. Epochs in which signal variations of EEG and EOG were larger than ± 100 μV were excluded from the averaging.

A negative component within 150–300 ms after each facial emotion change was evoked by all conditions for all groups at T5 (left), T5’ (2 cm below T5), T6 (right), and T6’ (2 cm below T6) electrodes in the left and right temporal areas. The peak latency and maximum amplitude for the adult group were significantly lower than those for the younger and older children groups. On the other hand, peak latency or maximum amplitude were not significantly different between the younger and older children.

In the younger children, the maximum amplitude of the negative component for N-H and N-A (appearance of each emotion) was significantly larger than those for H-N and A-N (disappearance of each emotion) in the right and left temporal area. On the other hand, in adults, the maximum amplitude of the negative component for N-H (appearance of positive emotion) was significantly larger than that for the other three conditions in the right temporal area. This pattern differed from that of younger and older children.

We found that the ERP pattern by facial emotional changes in 7–14-year-old children was different from that in adults for Japanese participants. This suggests that the facial emotional change perception process in face perception has not matured by age 14 in the Japanese population.

**Figure 1:**
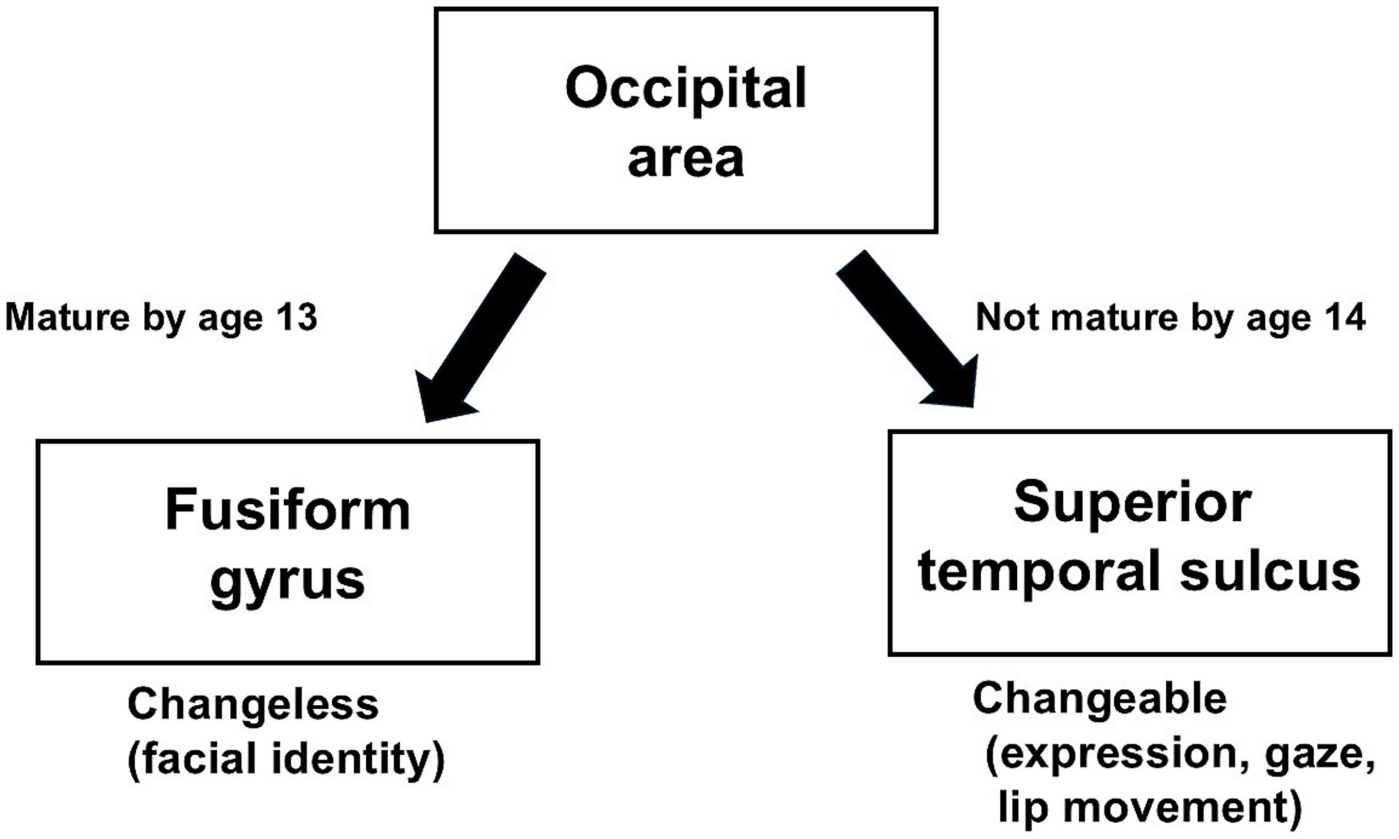
Summary of the two visual pathways for facial information that mature with development in the Japanese population.

In our two studies on ERP changes in face perception during development for Japanese participants, we hypothesized that the differing developmental patterns between face detection and facial emotional perception processes may be attributed to variations in the processing mechanisms associated with the two visual pathways for facial information within the model of the distributed human neural system for face perception (Haxby et al., 2000). Thus, we proposed the following hypothesis: the brain areas related to the process of facial changeless information, which involves the fusiform gyrus, are mature by age 13 in the Japanese population, whereas the brain areas related to the process of facial changeable information, which involves the superior temporal sulcus, are not mature by age 14 in the Japanese population (Figure 1).

Our two previous studies on developmental ERP changes related to face detection and facial emotional change perception for the Japanese population found that the maturation of these processes was independent of each other and differed from the patterns reported in studies of Western populations.

On the other hand, the age at which P300 matures in relation to face detection and emotional change perception during development in the Japanese population remains unclear. Future studies are needed to investigate the maturation of P300 evoked by face detection and emotional changes in Western and Japanese populations.

## Face perception by hospitality expertise in the Japanese population

Regarding our previous studies on the changes of face perception processes, we hypothesized that the ERPs related to face perception are affected by expertise. The Japanese population is considered to be good at hospitality compared with Western populations. The expression of Japanese hospitality is known as “OMOTENASHI.” Workers at Japanese hot spring spa inns are considered experienced and trained OMOTENASHI experts. Such workers are expected to observe guests carefully and respond to their state of mind.

In our study, we investigated whether ERPs related to facial emotion perception are affected by OMOTENASHI in Japanese participants (Miki et al., 2022). Forty Japanese females were divided into two groups: an OMOTENASHI (*n* = 21) group that consisted of workers at hot spring spa inns in Gamagori city, Aichi Prefecture, Japan, a well-known hot spring spa area; and a CONTROL (*n* = 19) group that had no experience in the hospitality, service, medical, or education fields. Three stimuli were presented to the participants: (1) Neutral: neutral face stimuli, (2) Happy: happy face stimuli, and (3) Angry: angry face stimuli. ERPs were recorded by averaging EEG. EEG electrodes were placed at Fz, Cz, T5, T6, Pz, O1, and O2 as well as T5’ (2 cm below T5) and T6’ (2 cm below T6), according to the international 10–20 system. The reference electrode was placed on the tip of the nose. The time frame of averaging was from 100 ms before and 400 ms after stimulus onset, with 100 ms before stimulus onset used as the baseline. Epochs in which signal variations of EEG and EOG were larger than ± 150 μV were excluded from the averaging.

The peak latency of P100 was not significantly different between the groups for each facial emotion stimulus; however, the maximum amplitude of P100 was significantly larger in the OMOTENASHI group than in the CONTROL group. In addition, the maximum amplitudes of P100 for neutral face at the right occipital area and angry face at the right and left occipital areas were significantly larger in the OMOTENASHI group than in the CONTROL group. On the other hand, there were no significant differences in the peak latency and maximum amplitude of N170 between the OMOTENASHI group and CONTROL group for each stimulus.

**Figure 2:**
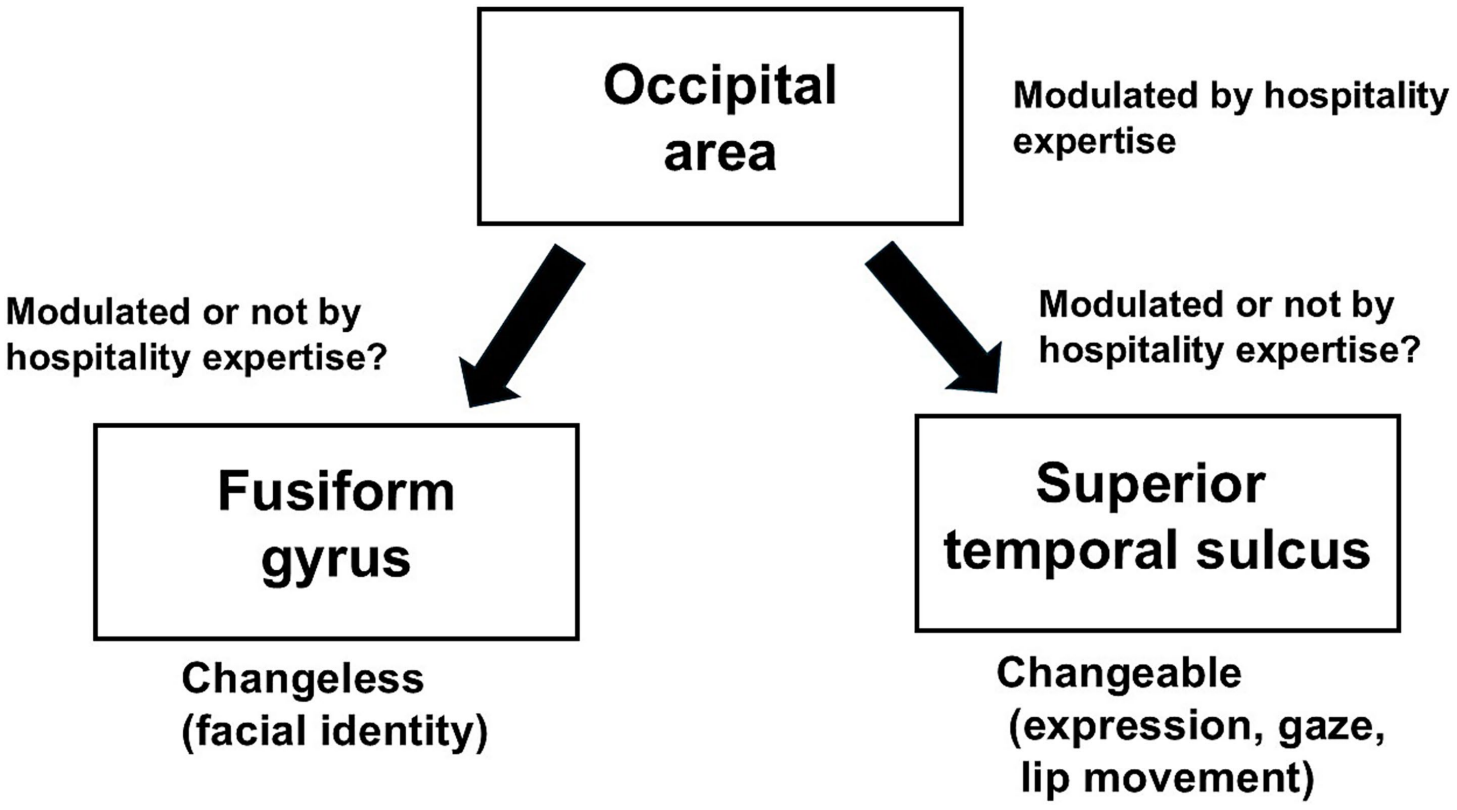
Summary of the two visual pathways for facial information modulated by hospitality expertise in the Japanese population.

Facial emotion perception may be affected by hospitality expertise in the Japanese population. We speculated that the training and experience involved in hospitality influence the early stages of the face perception process, such as the activity of the occipital area related to the early stage of the face perception process, in the Japanese population (Figure 2), which is due to automatic or subconscious attention to facial expressions. However, it is unclear whether the two visual pathways for facial information in the model of the distributed human neural system for face perception are influenced by hospitality expertise in the Japanese population (Figure 2). Therefore, studies related to the modulation of the two visual pathways for facial information in the Japanese population are needed.

## Conclusion

This review suggests the following responses to our starting hypotheses: (1) the age of maturation for face detection and facial emotional change perception processes are different in the Japanese population, (2) hospitality expertise due to training and experience may increase attention to emotion and affect the early stage of the face perception process in the Japanese population, and (3) the face perception process in the Japanese population differs from that in Western populations.

## References

[ref1] Allison T. Puce A. Spencer D. D. G M. C. (1999). Electrophysiological studies of human face perception. I: potentials generated in occipitotemporal cortex by face and non-face stimuli. Cereb. Cortex 9, 415–430. 10450888 10.1093/cercor/9.5.415

[ref2] Batty M. Taylor M. J. (2006). The development of emotional face processing during childhood. Dev. Sci. 9, 207–220. doi: 10.1111/j.1467-7687.2006.00480.x, PMID: 16472321

[ref3] Bentin S. Allison T. Puce A. Perez E. McCarthy G. (1996). Electrophysiological studies of face perception in humans. J. Cogn. Neurosci. 8, 551–565. doi: 10.1162/jocn.1996.8.6.551, PMID: 20740065 PMC2927138

[ref4] Bruce V. Young A. (1986). Understanding face recognition. Br. J. Psychol. 77, 305–327. doi: 10.1111/j.2044-8295.1986.tb02199.x 3756376

[ref5] Calder A. J. Young A. W. (2005). Understanding the recognition of facial identity and facial expression. Nat. Rev. Neurosci. 6, 641–651. doi: 10.1038/nrn1724, PMID: 16062171

[ref6] de Haan M. Johnson M. H. Halit H. (2003). Development of face-sensitive event-related potentials during infancy: a review. Int. J. Psychophysiol. 51, 45–58. doi: 10.1016/S0167-8760(03)00152-1, PMID: 14629922

[ref7] Dennis T. A. Malone M. M. Chen C. C. (2009). Emotional face processing and emotion regulation in children: an ERP study. Dev. Neuropsychol. 34, 85–102. doi: 10.1080/87565640802564887, PMID: 19142768 PMC2654398

[ref8] Farah M. J. Wilson K. D. Drain H. M. Tanaka J. R. (1995). The inverted face inversion effect in prosopagnosia: evidence for mandatory, face-specific perceptual mechanisms. Vis. Res. 35, 2089–2093. doi: 10.1016/0042-6989(94)00273-O 7660612

[ref9] George N. Evans J. Fiori N. Davidoff J. Renault B. (1996). Brain events related to normal and moderately scrambled faces. Brain Res. Cogn. Brain Res. 4, 65–76. doi: 10.1016/0926-6410(95)00045-3, PMID: 8883920

[ref10] Halit H. de Haan M. Johnson M. H. (2003). Cortical specialisation for face processing: face-sensitive event-related potential components in 3- and 12-month-old infants. NeuroImage 19, 1180–1193. doi: 10.1016/S1053-8119(03)00076-4, PMID: 12880843

[ref11] Haxby J. V. Hoffman E. A. Gobbini M. I. (2000). The distributed human neural system for face perception. Trends Cogn. Sci. 4, 223–233. doi: 10.1016/S1364-6613(00)01482-0, PMID: 10827445

[ref12] Hillyard S. A. Vogel E. K. Luck S. J. (1998). Sensory gain control (amplification) as a mechanism of selective attention: electrophysiological and neuroimaging evidence. Philos. Trans. R. Soc. Lond. Ser. B Biol. Sci. 353, 1257–1270. doi: 10.1098/rstb.1998.0281, PMID: 9770220 PMC1692341

[ref13] Hinojosa J. A. Mercado F. Carretié L. (2015). N170 sensitivity to facial expression: a meta-analysis. Neurosci. Biobehav. Rev. 55, 498–509. doi: 10.1016/j.neubiorev.2015.06.002, PMID: 26067902

[ref14] Hoehl S. Striano T. (2008). Neural processing of eye gaze and threat-related emotional facial expressions in infancy. Child Dev. 79, 1752–1760. doi: 10.1111/j.1467-8624.2008.01223.x 19037947

[ref15] Ishai A. (2008). Let's face it: it's a cortical network. NeuroImage 40, 415–419. doi: 10.1016/j.neuroimage.2007.10.040, PMID: 18063389

[ref16] Ishai A. Schmidt C. F. Boesiger P. (2005). Face perception is mediated by a distributed cortical network. Brain Res. Bull. 67, 87–93. doi: 10.1016/j.brainresbull.2005.05.027, PMID: 16140166

[ref17] Itier R. J. Alain C. Sedore K. McIntosh A. R. (2007). Early face processing specificity: it's in the eyes! J. Cogn. Neurosci. 19, 1815–1826. doi: 10.1162/jocn.2007.19.11.1815, PMID: 17958484

[ref18] Itier R. J. Latinus M. Taylor M. J. (2006). Face, eye and object early processing: what is the face specificity? NeuroImage 29, 667–676. doi: 10.1016/j.neuroimage.2005.07.041, PMID: 16169749

[ref19] Itier R. Taylor M. J. (2002). Inversion and contrast polarity reversal affect both encoding and recognition processes of unfamiliar faces: a repetition study using ERPs. NeuroImage 15, 353–372. doi: 10.1006/nimg.2001.0982, PMID: 11798271

[ref20] Itier R. J. Van Roon P. Alain C. (2011). Species sensitivity of early face and eye processing. NeuroImage 54, 705–713. doi: 10.1016/j.neuroimage.2010.07.031, PMID: 20650321 PMC3933319

[ref21] Jeon Y. W. Polich J. (2001). P3a from a passive visual stimulus task. Clin. Neurophysiol. 112, 2202–2208. doi: 10.1016/S1388-2457(01)00663-0, PMID: 11738190

[ref22] Kamps F. S. Richardson H. Murty N. A. R. Kanwisher N. Saxe R. (2022). Using child-friendly movie stimuli to study the development of face, place, and object regions from age 3 to 12 years. Hum. Brain Mapp. 43, 2782–2800. doi: 10.1002/hbm.25815, PMID: 35274789 PMC9120553

[ref23] Kiss M. Eimer M. (2008). ERPs reveal subliminal processing of fearful faces. Psychophysiology 45, 318–326. doi: 10.1111/j.1469-8986.2007.00634.x, PMID: 17995905 PMC2375009

[ref24] Kloth N. Itier R. J. Schweinberger S. R. (2013). Combined effects of inversion and feature removal on N170 responses elicited by faces and car fronts. Brain Cogn. 81, 321–328. doi: 10.1016/j.bandc.2013.01.002, PMID: 23485023 PMC3926862

[ref25] Leppänen J. M. Moulson M. C. Vogel-Farley V. K. Nelson C. A. (2007). An ERP study of emotional face processing in the adult and infant brain. Child Dev. 78, 232–245. doi: 10.1111/j.1467-8624.2007.00994.x, PMID: 17328702 PMC2976653

[ref26] Lin L. Wang C. Mo J. Liu Y. Liu T. Jiang Y.. (2020). Differences in behavioral inhibitory control in response to angry and happy emotions among college students with and without suicidal ideation: an ERP study. Front. Psychol. 11:543007. doi: 10.3389/fpsyg.2020.02191, PMID: 32982887 PMC7490336

[ref28] Mangun G. R. (1995). Neural mechanisms of visual selective attention. Psychophysiology 32, 4–18. doi: 10.1111/j.1469-8986.1995.tb03400.x, PMID: 7878167

[ref29] Meaux E. Hernandez N. Carteau-Martin I. Martineau J. Barthélémy C. Bonnet-Brilhault F.. (2014). Event-related potential and eye tracking evidence of the developmental dynamics of face processing. Eur. J. Neurosci. 39, 1349–1362. doi: 10.1111/ejn.12496, PMID: 24517386

[ref30] Melinder A. Gredebäck G. Westerlund A. Nelson C. A. (2010). Brain activation during upright and inverted encoding of own- and other-age faces: ERP evidence for an own-age bias. Dev. Sci. 13, 588–598. doi: 10.1111/j.1467-7687.2009.00910.x, PMID: 20590723 PMC2898522

[ref31] Miki K. Honda Y. Takeshima Y. Watanabe S. Kakigi R. (2015). Differential age-related changes in N170 responses to upright faces, inverted faces, and eyes in Japanese children. Front. Hum. Neurosci. 9:263. doi: 10.3389/fnhum.2015.00263, PMID: 26082700 PMC4451338

[ref32] Miki M. Takeshima Y. Kida T. Kakigi R. (2022). The ERP and psychophysical changes related to facial emotion perception by expertise in Japanese hospitality, “OMOTENASHI”. Sci. Rep. 12:9089. 35701462 10.1038/s41598-022-11905-2 PMC9197832

[ref34] Miki K. Watanabe S. Teruya M. Takeshima Y. Urakawa T. Hirai M.. (2011). The development of the perception of facial emotional change examined using ERPs. Clin. Neurophysiol. 122, 530–538. doi: 10.1016/j.clinph.2010.07.013, PMID: 20724212

[ref35] Nemrodov D. Anderson T. Preston F. F. Itier R. J. (2014). Early sensitivity for eyes within faces: a new neuronal account of holistic and featural processing. NeuroImage 97, 81–94. doi: 10.1016/j.neuroimage.2014.04.042, PMID: 24768932 PMC5321665

[ref36] Nemrodov D. Itier R. J. (2011). The role of eyes in early face processing: a rapid adaptation study of the inversion effect. Br. J. Psychol. 102, 783–798. doi: 10.1111/j.2044-8295.2011.02033.x, PMID: 21988384 PMC3933317

[ref37] Peykarjou S. Hoehl S. (2013). Three-month-olds' brain responses to upright and inverted faces and cars. Dev. Neuropsychol. 38, 272–280. doi: 10.1080/87565641.2013.786719, PMID: 23682666

[ref38] Rossignol M. Philippot P. Douilliez C. Crommelinck M. Campanella S. (2005). The perception of fearful and happy facial expression is modulated by anxiety: an event-related potential study. Neurosci. Lett. 377, 115–120. doi: 10.1016/j.neulet.2004.11.091, PMID: 15740848

[ref39] Rossion B. Delvenne J. F. Debatisse D. Goffaux V. Bruyer R. Crommelinck M.. (1999). Spatio-temporal localization of the face inversion effect: an event-related potentials study. Biol. Psychol. 50, 173–189. doi: 10.1016/S0301-0511(99)00013-7 10461804

[ref40] Rossion B. Gauthier I. Tarr M. J. Despland P. Bruyer R. Linotte S.. (2000). The N170 occipito-temporal component is delayed and enhanced to inverted faces but not to inverted objects: an electrophysiological account of face-specific processes in the human brain. Neuroreport 11, 69–74. doi: 10.1097/00001756-200001170-00014, PMID: 10683832

[ref41] Rossion B. Jacques C. (2008). Does physical interstimulus variance account for early electrophysiological face sensitive responses in the human brain? Ten lessons on the N170. NeuroImage 39, 1959–1979. doi: 10.1016/j.neuroimage.2007.10.011, PMID: 18055223

[ref42] Sadeh B. Yovel G. (2010). Why is the N170 enhanced for inverted faces? An ERP competition experiment. NeuroImage 53, 782–789. doi: 10.1016/j.neuroimage.2010.06.029, PMID: 20558303

[ref43] Safar K. Moulson M. C. (2020). Three-month-old infants show enhanced behavioral and neural sensitivity to fearful faces. Dev. Cogn. Neurosci. 42:100759. doi: 10.1016/j.dcn.2020.100759, PMID: 32072932 PMC7015984

[ref44] Schindler S. Bruchmann M. Gathmann B. Moeck R. Straube T. (2021). Effects of low-level visual information and perceptual load on P 1 and N170 responses to emotional expressions. Cortex 136, 14–27. doi: 10.1016/j.cortex.2020.12.011, PMID: 33450599

[ref45] Shibata T. Nishijo H. Tamura R. Miyamoto K. Eifuku S. Endo S.. (2002). Generators of visual evoked potentials for faces and eyes in the human brain as determined by dipole localization. Brain Topogr. 15, 51–63. doi: 10.1023/A:1019944607316, PMID: 12371677

[ref46] Taylor M. J. Batty M. Itier R. J. (2004). The faces of development: a review of early face processing over childhood. J. Cogn. Neurosci. 16, 1426–1442. doi: 10.1162/0898929042304732, PMID: 15509388

[ref47] Taylor M. J. Edmonds G. E. McCarthy G. Allison T. (2001a). Eyes first! Eye processing develops before face processing in children. Neuroreport 12, 1671–1676. doi: 10.1097/00001756-200106130-00031, PMID: 11409737

[ref48] Taylor M. J. Itier R. J. Allison T. Edmonds G. E. (2001b). Direction of gaze effects on early face processing: eyes-only versus full faces. Brain Res. Cogn. Brain Res. 10, 333–340. doi: 10.1016/S0926-6410(00)00051-3, PMID: 11167057

[ref49] Vlamings P. H. J. M. Jonkman L. M. Kemner C. (2010). An eye for detail: an event-related potential study of the rapid processing of fearful facial expressions in children. Child Dev. 81, 1304–1319. doi: 10.1111/j.1467-8624.2010.01470.x, PMID: 20636697

[ref50] Xie W. McCormick S. A. Westerlund A. Bowman L. C. Nelson C. A. (2019). Neural correlates of facial emotion processing in infancy. Dev. Sci. 22:e12758. doi: 10.1111/desc.12758, PMID: 30276933 PMC6443490

